# Contribution of Endoscopy in the Diagnosis and Treatment of Patients With Primary Sclerosing Cholangitis

**DOI:** 10.1111/den.15074

**Published:** 2025-07-02

**Authors:** Mamoru Takenaka, Masatoshi Kudo

**Affiliations:** ^1^ Department of Gastroenterology and Hepatology Kindai University Faculty of Medicine Osaka‐Sayama Japan

Primary sclerosing cholangitis (PSC) is an autoimmune liver and biliary disease characterized by fibrotic narrowing of the bile ducts, leading to chronic bile stasis and eventually progressing to cirrhosis. However, to date, the etiology of PSC remains unknown.

A diagnosis of PSC is made based on the following major findings: typical bile duct images and elevated serum alkaline phosphatase level; minor findings include the presence of inflammatory bowel disease (IBD) as well as histopathological findings of the liver [1, 2]. Regarding the treatment, liver transplantation is the only curative treatment, and ursodeoxycholic acid and bezafibrate are used as symptomatic treatments.

It means that endoscopy is not essential for the diagnosis and treatment of PSC; however, there are situations where endoscopic findings or treatment can contribute.

However, because endoscopic intervention for patients with PSC involves a high risk, appropriate patient selection is extremely important, so it is necessary to have a deep knowledge of the findings on the contribution of endoscopes to PSC to date.

In the latest issue of *Digestive Endoscopy*, Mizuno et al. presented an in‐depth and interesting review, titled “Endoscopic management of primary sclerosing cholangitis” [3].

In this article, we discuss the contribution of endoscopic examinations in patients with PSC in light of this review.

## 1 The Contribution of Endoscopy to the Diagnosis of PSC

Typical bile duct images are a key factor in the diagnosis of PSC. For a long time, bile duct imaging has been carried out using endoscopic retrograde cholangiography (ERC); however, it is impossible to inject contrast medium into all of the bile ducts, so it is impossible to evaluate the degree of all bile ducts. In addition, in cases where many bile duct strictures exist or the strictures are severe, there remains a risk of the contrast medium not being excreted, leading to postprocedural cholangitis or pancreatitis. Further, the management of cholangitis that occurs in conjunction with PSC can be extremely challenging. In addition, the corresponding radiation exposure not only affects endoscopists but also medical staff; thus, performing ERC without actual necessity is unacceptable [4, 5].

Magnetic resonance cholangiopancreatography (MRCP) has been reported to have both high diagnostic sensitivity and specificity and is recommended as the first‐line diagnostic method for PSC [1, 2, 3]; thus, it is difficult to justify performing ERC for the sole purpose of cholangiography.

The greatest contribution of ERC to the diagnosis of PSC is that it can be used to obtain tissue samples for differentiation of PSC from bile duct cancer. The findings of bile duct stenosis in PSC are sometimes very similar to those in bile duct cancer. Moreover, it is reported that patients with PSC are at risk of developing bile duct cancer, with cumulative risks of 6%, 14%, and 20% at 10, 20, and 30 years of PSC, respectively [6]. Therefore, obtaining tissue samples from the site of bile duct stenosis can be of great benefit in patients with PSC. However, the sensitivity of brush cytology, which is performed for tissue sampling, is low, and to improve the diagnostic accuracy, repeated brush cytology becomes necessary.

Intraductal ultrasonography (IDUS) can assess bile duct wall thickening and is useful for distinguishing between PSC, IgG4‐related cholangitis (IgG4‐SC), and bile duct cancer. Asymmetric thickening and intermittent changes suggest bile duct cancer, while symmetric and smooth thickening suggest IgG4‐related cholangitis (IgG4‐SC). Asymmetric and irregular wall thickening with unclear borders may indicate PSC.

Endoscopic ultrasound (EUS) can perform the same detailed evaluation of bile duct wall thickening as IDUS, without radiation exposure. Furthermore, it can also evaluate lymph node enlargement, contributing to the diagnosis of PSC.

Peroral cholangioscopy (POCS) is useful for differentiating PSC from bile duct cancer, as it allows observation of the mucosal structure of the bile duct wall, enables tissue biopsy, and is useful for evaluating the degree of bile duct inflammation [7]. During active inflammation, erythema of the mucosa, ulcers, fibrinous white exudate, and irregular surfaces can be observed. On the other hand, in cases of chronic inflammation, scarring, pseudodiverticula, and bile duct strictures may be observed. These characteristics not only enable the diagnosis of PSC but also the classification of the disease stage and may be useful for evaluating the response to drug therapy.

However, in severe cases of stenosis, it may be impossible to insert a POCS into a bile duct with multiple stenoses or a severely narrowed bile duct. Therefore, it is hoped that the development of thin‐diameter POCS will contribute to PSC.

Furthermore, considering the importance of IBD status in diagnosing PSC, colonoscopy is recommended for cases suspected of PSC. In Japan, colonoscopy is not sufficiently performed for evaluating IBD in PSC patients, and it should be recognized that colonoscopy is one of the endoscopic examinations that can contribute to the diagnosis of IBD.

## 2 The Contribution of Endoscopy to the Treatment of PSC

Liver transplantation is the only curative treatment for PSC. However, in countries such as Japan, where brain‐dead donors are few and several living‐donor liver transplantations are performed, this problem is particularly glaring, as endoscopic treatment can avoid liver transplantation or delay the need for it. In clinical practice, endoscopic intervention is performed on patients with bacterial cholangitis who do not respond to antibiotic treatment.

The main endoscopic treatments for PSC include “endoscopic balloon dilation for stricture” and “drainage treatment.” The review by Mizuno et al. provides a good summary of these two endoscopic techniques and summarizes the key points of each technique [3].

With regard to endoscopic balloon dilation for PSC related strictures, a prospective study reported the usefulness of stepwise dilation of the bile duct. In patients who respond well to initial balloon dilation, this procedure is considered for repeated use.

In a retrospective study, it has been reported that planned balloon dilation results in a higher survival rate without the need for transplantation in patients compared to those who undergo dilation on demand after the onset of symptoms [8].

However, there is no consensus on the dilatation protocol or the optimal balloon size, and further research is needed for repeating procedures. So, at present, actively performing balloon dilatation in asymptomatic patients is not recommended.

Several reports have shown the effectiveness of plastic stent placement for PSC, and it is recommended that one 10‐Fr stent should be placed for extrahepatic stenosis and two 7‐Fr stents should be placed for hilar stenosis; these should then be replaced after 1–4 weeks to take into account the possibility of early stent occlusion [9].

In a retrospective study comparing balloon dilation alone with balloon dilation plus stent placement, the combined therapy group required more procedures, resulting in a higher complication rate [3]. Further, a multicenter randomized trial was discontinued early because of a high risk of serious adverse events, such as bacterial cholangitis and pancreatitis, in the stent group [3]. A recent meta‐analysis reported that patients who underwent balloon dilation alone had a higher technical success rate, clinical success rate, and a lower complication rate [10].

Therefore, balloon dilation is now considered superior to stent placement for the treatment of strictures in patients with PSC.

In Western guidelines, the choice between balloon dilation and stent placement is left to the discretion of the endoscopist, but based on these results, the Japanese guidelines recommend balloon dilation for patients with PSC [1].

However, in actual clinical practice, there are cases in which clinical improvement is not achieved with balloon dilation alone, and in such cases, PS placement is often useful.

Endoscopists treating PSC must fully understand the advantages and disadvantages of both balloon dilation and PS placement and strive to provide appropriate treatment for each case.

## 3 Future Developments

There have been reports on the use of fluorescence in situ hybridization analysis and confocal laser endoscopy for the diagnosis of PSC, and both have shown high bile duct cancer diagnostic capabilities; however, further research should be conducted to confirm the usefulness of methods other than brush cytology [3].

At present, the usefulness of metal stents in the treatment of biliary drainage for PSC has not been verified. However, the effectiveness of metal stents for benign bile duct stenosis has been proven, and further verification is needed to determine whether they are also useful for PSC [3].

Several reports have shown that endoscopic ultrasound‐guided tissue acquisition is useful for differentiating between PSC and bile duct cancer or IgG4‐related sclerosing cholangitis, but whether it can really contribute to the diagnosis depends on further research [3].

EUS‐guided biliary drainage (EUS‐BD) was developed as a rescue procedure in cases where papillary drainage was difficult. However, due to the diffuse nature of the disease, its effectiveness in patients with PSC is thought to be limited; nevertheless, EUS‐BD may be beneficial in some cases [3].

In summary, endoscopic examinations can make a certain contribution to patients with PSC. Histological examination is useful for differentiating PSC from cancer, and endoscopic treatments, such as balloon dilation and biliary drainage, are effective. However, it is essential to be aware of the high risk of complications when performing endoscopy for PSC, and careful patient selection is essential.

Patients and their families should be fully informed of the risks involved in each procedure, and informed consent should be obtained. The procedure should be performed by an experienced endoscopist who is familiar with the unique issues of PSC and who can take appropriate decisions for management and treatment.

## Author Contributions

Mamoru Takenaka drafted the manuscript, and Masatoshi Kudo critically revised the manuscript for important intellectual content.

## Conflicts of Interest

All authors declare no conflicts of interest. M.T. is an AE of *Digestive Endoscopy*.

## Linked Articles

Endoscopic management of primary sclerosing cholangitis. https://doi.org/10.1111/den.15010.
